# Treating Facial Scars using Polydioxanone Threads

**DOI:** 10.1016/j.jpra.2025.01.006

**Published:** 2025-01-15

**Authors:** Mohammad Khaled Hamolaila, Mazen Zenati, Asaad Shehada

**Affiliations:** Department of oral and maxillofacial surgery, Damascus university, Damascus, Syria

**Keywords:** Atrophic scars, Polydioxanone (PDO) threads, non-surgical techniques

## Abstract

Traditional scar treatments, such as laser therapy, chemical peels, and surgery, are expensive and require long recovery times. Polydioxanone (PDO) threads offer a minimally invasive and cost-effective solution that enhances collagen production and skin texture.

This study aimed to evaluate the effectiveness of PDO threads in the management of atrophic facial scars due to the lack of clinical research on this topic.

A prospective clinical study was conducted on 20 patients with facial atrophic scars caused by accidents or previous surgical procedures. The patient and observer scar assessment scale was used to evaluate the scars from the observer's and patient's perspectives at three-time points for each patient. The observer assessment included the following variables: vascularity, pigmentation, thickness, pliability, surface area, and homogeneity. The patient assessment included the following variables: pain, color, stiffness, thickness, appearance, and itching**.**

Statistically significant improvement was observed in atrophic facial scars treated using PDO threads in all observer variables (p<0.001). Significant improvement was recorded in the patient's color, stiffness, thickness, and appearance variables; however, itching sensation increased between T0 and T1 with no statistically significant differences in the pain variable.

Within the limits of this study, we conclude that PDO threads are known for ease of use, availability, and biocompatibility. They biodegrade naturally, reducing irritation risk. By stimulating collagen, PDO threads promote natural skin regeneration and thereby improve scars without using foreign materials. The treatment method is safe and minimally invasive, with short recovery times. Clinical studies showed significant improvements in atrophic facial scars with high patient satisfaction.

## Introduction

Wound healing is a complex and highly regulated biological process that is fundamentally divided into 3 overlapping phases: the inflammatory, cellular proliferation, tissue remodeling phases, and scars form during the final phase of skin-wound healing (1).

Under normal physiological conditions, the young scar undergoes maturation over several months, involving tissue remodeling within the scar. This process is associated with a reduction in cellular inflammation, blood vessels, collagen fibers, and fibroblasts. However, the inflammatory activity can persist longer by hindering the scar maturation process and consequently retaining the scar in its immature phase for an extended period. This results in the formation of pathological scars (2,3).

The primary goal in managing visible pathological scars is to improve aesthetics, boost the patient's self-confidence, and enhance their quality of life, in addition to alleviating the symptoms, such as pain, discomfort and restricted skin movement, associated with the scars (4).

Corrective procedures for managing skin scars include non-invasive techniques characterized by their ease of application and minimal discomfort for the patient, and surgical invasive techniques for excising and removing pathological scars along with the surrounding damaged tissues (5).

Non-invasive techniques, such as laser therapy and medical needling, offer benefits including lower risks of complications and shorter recovery times, making them ideal for mild to moderate scars. However, they may require multiple sessions to achieve optimal results and might not be effective in treating severe scars. Contrastingly, invasive techniques, including scar revision and excision, provide immediate results and are suitable for more pronounced scars; however, they carry risks such as infection and longer recovery periods (6).

Polydioxanone (PDO), commercially known as monofilament threads under the brand name PDS®, was introduced by Ethicon in 1981 and received approval from the US Food and Drug Administration under number K190264. Compared to polypropylene threads, PDO threads offer greater elasticity and tensile strength while eliciting milder tissue reactions and being safely absorbed via hydrolysis (7). Although they are used commonly in cosmetic procedures to enhance skin quality and reduce signs of aging, they have not achieved considerable recognition in scar management, despite their healing properties and role as a dermal filler.

In addition to PDO, several other suture materials have been used in wound management, each having distinct properties. Polypropylene, for instance, is a non-absorbable material known for its high tensile strength and resistance to infection, but it may cause more tissue irritation than PDO. Although polyglycolic acid and polyglactin 910 (Vicryl®) are absorbable materials that promote wound healing with minimal inflammatory response, they may degrade more quickly in high-tension areas. Silk, despite being easy to handle and offering good knot security, has been largely replaced by synthetic materials due to its potential for developing infection and degrading slowly (8).

## Objectives

Therefore, using PDO threads represents an easy, cost-effective, and viable method for scar management. However, relatively few studies have documented their efficacy in scar improvement compared to the extensive literature on their effectiveness in reducing signs of aging. This study presents the management of patients with facial atrophic scars using PDO threads and follows up on their outcomes at 6 months post-treatment.

## Material and Methods

A prospective clinical study was conducted on 20 patients with facial atrophic scars caused by previous facial surgical procedures or accidents. The study aimed to evaluate the effectiveness of PDO threads in managing these scars. The study was conducted on healthy adults in the Department of Oral and Maxillofacial Surgery at the Faculty of Dentistry, Damascus University.

Sample size estimation was based on a similar clinical study (9). GPower 3.1 software was used to determine the minimum sample size, and the minimum sample size was found to be 14 samples. The total size was increased to 20 samples.

Patients meeting the following criteria were included in the research sample: those with linear atrophic facial scars from previous surgical procedures or prior accidents, with at least 2-cm long scar and age ranging from 18 to 40 years. Additionally, scars should be at least 6 months old, and the patients should be capable of adhering to the research protocol and completing the follow-up sessions.

At the preoperative phase, surgical site examination was conducted to assess any abnormalities, determine the dimensions of the scar, identify its cause and duration since onset, and review the patient's previous scar treatment history. Based on the aforementioned clinical diagnostic data, eligible patients were enrolled based on the inclusion criteria. Each patient was provided with information about the study, the surgical procedure, potential complications, and the specified follow-up appointments. Informed consent was obtained for the surgical procedure, and photographs of the patient were taken before the procedure to evaluate the scar.

At the surgical phase, skin sterilization at the surgical site was performed using 4% povidone iodine solution, followed by skin anesthesia, which achieved with 20% benzocaine typical anesthetic. Local anesthesia at the surgical site was also administered through the injection of 2% lidocaine containing 1:80,000 epinephrine along the edges of the scar. Using an 18-gauge needle, a point of entry was created at the beginning of the scar and the needle was inserted along the length of the scar to its end, creating a pathway for inserting the PDO-carrying threads. The needles carrying the threads (with non-working heads) and loaded with 14 threads were then inserted. PDO threads (BeauMed, Hydra Multi, Korea) measuring 50 mm (thread) and 38 mm (needle) were used. These threads were smooth and had a non-working head needle containing 14 PDO threads of size 0-7, with a core thread size of 0-5. The subcutaneous layer was the target layer for placing the threads, the cannula was inserted, and with gentle application of pressure by the left hand on the scar, the cannula (needle carrying the threads) was smoothly removed, leaving the threads in place under the skin and trimming the excess portion of the threads.

At the post-operative phase, the patient was given the following instructions: Apply ice packs to the scar for 10 min, limit facial movement for 24 h, and avoid facial creams or cosmetics for 48 h. Refrain from facial pulling or massaging for 2 weeks and avoid strenuous exercise for 3 days. The patient was advised to take pain medication only when necessary and if experiencing discomfort.

The patient and observer scar assessment scale (POSAS) (10) was used to evaluate the scars from the observer's and patient's perspectives at three-time points for each patient: T0 (at the time of the procedure), T1 (one month after the procedure), and T2 (6 months after the procedure). The observer's assessment included the following variables: vascularity, pigmentation, thickness, pliability, surface area, and homogeneity. Each variable was scored from 1 (completely normal skin) to 10 (worst possible scar). The patient's assessment included the following variables: pain, color, stiffness, thickness, appearance, and itching, each one was scored from 1 (not at all) to 10 (too much).

For the statistical analysis, software SPSS v. 20.0 was used. A p-value <0.05 was deemed as statistically significant. Significant statistical differences between the study times were determined using the paired t-test. After investigating the normal distribution of the study variables using the Kolmogorov–Smirnov test, the descriptive statistical analysis was carried out.

## Results

The study included 20 patient, with age ranging between 18 to 47 years with a mean 27.2±9.8 years. The study sample consisted of 75% women and 25% men. Table 1 shows the descriptive statistics of the POSAS at T0, T1, and T2 from the observer's perspective. Table 2 shows the statistically significant differences between the studied time periods (at the time of the procedure, one month after the procedure, and 6 months after the procedure) in the sample. This indicates that there is an improvement in the scar in terms of vascularity, pigmentation, thickness, pliability, surface area, and homogeneity from the observer's perspective.

**Table 1:** Descriptive statistics of the patient and observer scar assessment scale at T0, T1, and T2 from the observer's perspective.

Studied variable	Time period	Minimum	Maximum	Mean	Standard deviation
Vascularity	T0	3.67	7.33	5.70	1.04
	T1	2.00	5.67	3.95	0.96
	T2	1.00	4.33	2.87	0.84
Pigmentation	T0	3.67	7.67	5.68	0.96
	T1	2.33	6.00	3.93	0.83
	T2	1.33	3.67	2.52	0.77
Thickness	T0	4.67	7.67	6.13	0.85
	T1	2.33	5.33	3.85	0.77
	T2	2.00	3.33	2.48	0.40
Pliability	T0	3.67	8.00	5.92	1.11
	T1	2.67	5.33	3.65	0.60
	T2	1.33	3.67	2.47	0.53
Surface Area	T0	4.00	6.67	5.52	0.81
	T1	2.67	5.00	3.95	0.69
	T2	1.67	4.33	2.90	0.58
Homogeneity	T0	4.33	7.67	5.82	1.09
	T1	2.67	5.33	3.85	0.66
	T2	1.67	4.33	2.65	0.67

**Table 2:** Paired t- test between the studied time periods from the observer's perspective.

Studied variable	Comparison between the two time periods	t-value	p-value
Vascularity	T1-T0	-11.319	0.001>
	T2-T0	-16.170	0.001>
	T2-T1	-8.337	0.001>
Pigmentation	T1-T0	-8.596	0.001>
	T2-T0	-14.330	0.001>
	T2-T1	-11.517	0.001>
Thickness	T1-T0	-11.427	0.001>
	T2-T0	-17.979	0.001>
	T2-T1	-8.434	0.001>
Pliability	T1-T0	-10.572	0.001>
	T2-T0	-15.359	0.001>
	T2-T1	-11.706	0.001>
Surface Area	T1-T0	-9.542	0.001>
	T2-T0	-14.174	0.001>
	T2-T1	-7.764	0.001>
Homogeneity	T1-T0	-9.098	0.001>
	T2-T0	-14.330	0.001>
	T2-T1	-6.921	0.001>

Table 3 shows the descriptive statistics of the POSAS at T0, T1, and T2 from the patient's perspective. Table 4 shows statistically significant differences between the studied time periods (at the time of the procedure, one month after the procedure, and 6 months after the procedure) in the research sample. This suggests that there was an improvement in the scar in terms of color, stiffness, thickness, and appearance from the patient's perspective. However, itching sensation increased during the first month after the procedure and decreased over 6 months. There were no statistically significant differences in the pain variable.

**Table 3:** Descriptive statistics of the patient and observer scar assessment scale at T0, T1, and T2 from the patient's perspective.

Studied variable	Time period	Minimum	Maximum	Mean	Standard deviation
Pain	T0	1	1	1.00	0
	T1	1	4	1.35	0.88
	T2	1	1	1.00	0
Itchiness	T0	1	5	1.50	1.28
	T1	1	4	2.40	1.31
	T2	1	2	1.20	0.41
Skin color	T0	4	8	5.75	1.02
	T1	2	5	3.55	1.00
	T2	1	4	2.55	0.94
Hardness	T0	4	8	5.65	1.27
	T1	2	5	3.45	0.94
	T2	1	4	2.20	1.01
Thickness	T0	3	8	5.75	1.33
	T1	2	6	3.55	1.15
	T2	1	6	2.70	1.08
Appearance	T0	7	10	8.25	0.97
	T1	3	6	4.55	1.05
	T2	1	4	2.50	0.95

**Table 4:** Paired t -test results between the studied time periods from the patient's perspective.

Studied variable	Comparison between the two time periods	t-value	p-value
Pain	T1-T0	1.789	0.090
	T2-T0	-	-
	T2-T1	-1.789	0.090
Itchiness	T1-T0	2.349	0.030
	T2-T0	-1.064	0.301
	T2-T1	-4.188	0.001>
Skin color	T1-T0	-11.000	0.001>
	T2-T0	-10.514	0.001>
	T2-T1	-4.359	0.001>
Hardness	T1-T0	-7.228	0.001>
	T2-T0	-10.262	0.001>
	T2-T1	-5.784	0.001>
Thickness	T1-T0	-8.223	0.001>
	T2-T0	-7.616	0.001>
	T2-T1	-3.847	0.001
Appearance	T1-T0	-12.333	0.001>
	T2-T0	-19.294	0.001>
	T2-T1	-9.706	0.001>

Table 5 shows the descriptive statistics for the over-all mean for variables from the patient's and observer's perspective with the independent t-test results comparing the gender groups. Table 6 shows the descriptive statistics for the over-all mean for variables from the patient's and observer's perspective with the independent t-test results comparing the age groups.

**Table 5:** Descriptive statistics for over-all variable and independent t- test results between the genders.

Studied variable	Time period	Gender	N	Mean	t-value	p-value
Over-all *from the observer's perspective*	T0	Male	5	5.98	0.528	0.604
		Female	15	5.73		
	T1	Male	5	3.76	-0.154	0.879
		Female	15	3.80		
	T2	Male	5	2.58	-1.199	0.246
		Female	15	2.93		
Over-all from the patient's perspective	T0	Male	5	4.78	1.661	0.114
		Female	15	4.27		
	T1	Male	5	3.18	0.350	0.743
		Female	15	3.03		
	T2	Male	5	2.01	-0.255	0.840
		Female	15	2.07

**Table 6:** Descriptive statistics for over-all variable and independent t- test results between the age groups.

Studied variable	Time period	Age groups	N	Mean	t-value	p-value
Over-all *from the observer's perspective*	T0	30>	14	6.02	0.825	0.420
		30<	6	5.67		
	T1	30>	14	3.86	1.174	0.256
		30<	6	3.56		
	T2	30>	14	2.71	0.550	0.589
		30<	6	2.56		
Over-all from the patient's perspective	T0	30>	14	4.71	0.698	0.494
		30<	6	4.50		
	T1	30>	14	3.18	0.478	0.639
		30<	6	3.06		
	T2	30>	14	2.07	0.774	0.449
		30<	6	1.92		

## Discussion

Intact skin acts as a crucial defense against external elements and proper wound healing is essential for tissue stability restoration. Although scarring is a natural process, it can cause unaesthetic results that are particularly impactful in prominent areas such as the face, affecting the psychological well-being of the patient (11,12).

Treatment approaches for skin scars vary based on their characteristics. Techniques aimed at removing excess tissue, such as topical injections and surgery, are employed for hypertrophic scars with raised surfaces. Conversely, for atrophic scars with depressed surfaces, methods such as dermabrasion and application of tissue fillers such as hyaluronic acid and autologous fat are commonly employed. In recent years, highly elastic absorbable threads, such as PDO threads, have emerged as a treatment options for addressing volume deficits in atrophic scars; these threads are notable for their durability under pressure on the treated skin and their ability to promote inflammatory and immune responses, which reduce the risk of complications and infections (9,13).

PDO threads are available in smooth or barbed forms. Smooth threads are preferred for facial treatments owing to their ease of application and ability to fill tissue while minimizing tissue damage. This study specifically focused on smooth PDO threads, which act as fillers to increase tissue volume and compensate for atrophic scars (14,15).

Our research included a clinical study to evaluate the improvement of scars treated with PDO threads after the follow-up period. The POSAS score after 6 months was lower, indicating a clear and statistically significant improvement in atrophic facial scars treated using these threads.

Considering the rarity of the clinical studies evaluating the efficacy of PDO threads in treating atrophic facial scars, we interpreted the results of our study within the context of the available published clinical research.

A study involving 23 patients assessed the efficacy of PDO threads in treating atrophic neck scars. The PDO threads were used to address tissue defects within the scars, leading to noticeable improvement in scar appearance and patient satisfaction after 6 months after treatment. The study findings indicated that PDO thread usage enhances cosmetic outcomes, aids in scar healing, boosts patient satisfaction, and diminishes the necessity for corrective surgery to address scar defects (16).

A study compared the efficacy of poly-L-lactic acid (PLLA) threads with hyaluronic acid in treating atrophic facial scars. Forty patients were treated using the threads and those treated with PLLA threads showed notable clinical improvement rate of 82.4% after 6 months (17).

A study compared PDO monofilament threads with carbon dioxide laser in treating atrophic facial scars. Ten patients received laser treatment on the right side of the face and PDO threads on the left. The follow-up lasted 3 months during which three-dimensional scanning was carried out. Both methods improved facial aesthetics and skin texture in the treated areas. However, the researchers favored the carbon dioxide laser owing to its ease of use. Furthermore, they acknowledged that PDO threads were more cost-effective for the patients (18).

**Figure:**
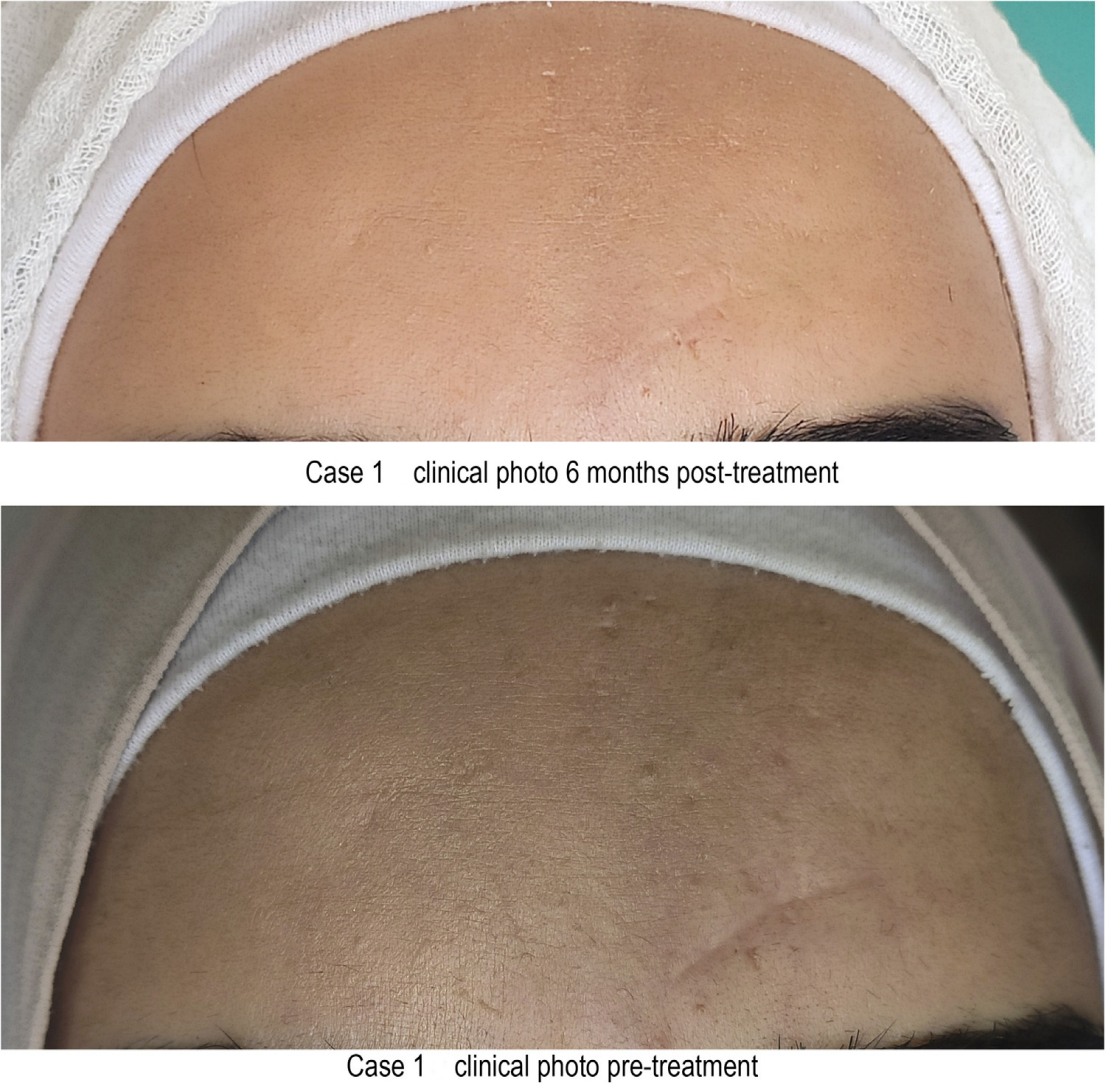

**Figure:**
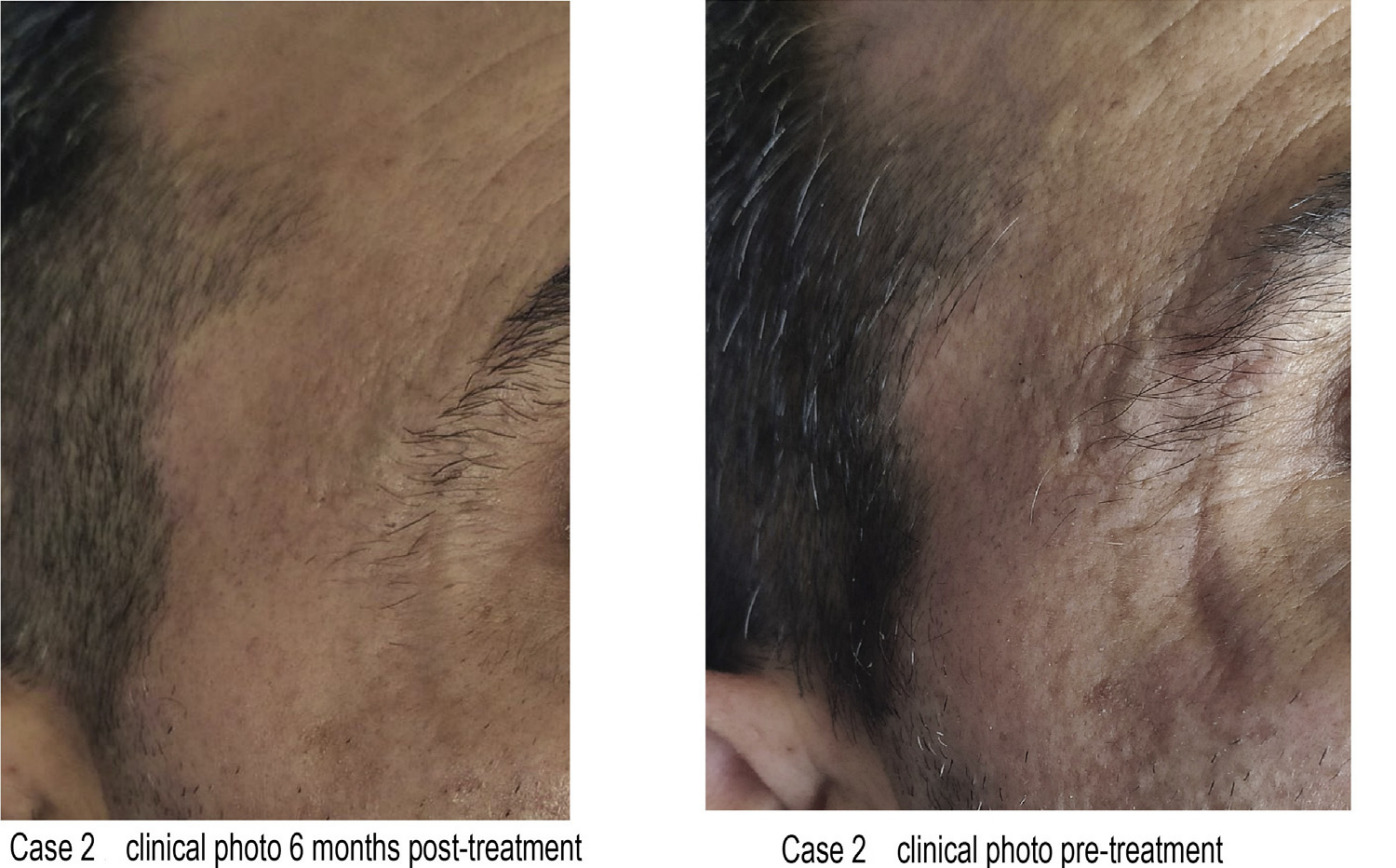

## Conclusion

Within the limits of the present study, it can be concluded that PDO threads are known for their ease of use, availability, and biocompatibility. These threads are biodegradable and absorbed by the body, reducing the risks of irritation or rejection. By stimulating collagen production, PDO threads aid in skin regeneration and gradually improve the appearance of scars by leveraging the skin's natural processes. Furthermore, they provide outcomes that appear natural without using foreign materials or fillers. Clinical studies have reported evident improvements in managing atrophic facial scars with high patient satisfaction, when these scars are treated with PDO threads. PDO threads are considered safe and minimally invasive, leading to shorter recovery times and fewer side effects compared to methods such as surgical procedures or autologous fat injections.

## Conflict of interest

All authors have declared no conflicts of interest.
